# Excessive use of social media by high school students in southern Brazil

**DOI:** 10.1590/1984-0462/2022/40/2020420IN

**Published:** 2022-05-27

**Authors:** Yohana Pereira Vieira, Vanise dos Santos Ferreira Viero, Elizabet Saes-Silva, Priscila Arruda da Silva, Laura Silva da Silva, Mirelle de Oliveira Saes, Lauro Miranda Demenech, Samuel Carvalho Dumith

**Affiliations:** a Universidade Federal do Rio Grande, Rio Grande, RS, Brasil.

**Keywords:** Adolescent, Social Network Use, Risk behavior, Cross sectional studies, Adolescente, Rede social, Comportamento de risco, Estudos transversais

## Abstract

**Objective:**

To assess the prevalence of excessive use of social media and associated factors, as well as possible health consequences in high school students in southern Brazil.

**Methods:**

This is a population-based cross-sectional study, conducted with high school students in the city of Rio Grande, RS. All students who were attending high school at the Federal Institute of Rio Grande do Sul, *campus* Rio Grande, were eligible for this research in the second semester of 2019. In total, 513 students participated in the study. The dependent variable was excessive use of social media, defined as more than five hours per day. Descriptive and bivariate analyses were carried out and the Poisson regression was used to verify associations, with robust adjustment of variance.

**Results:**

The prevalence of students who reported excessive use of social media was 35.9%. The groups that were most susceptible to excessive use of social media had the following profile: female, black/brown skin, aged between 18 and 20 years old, attending the first year of high school. Excessive use of social media was shown to be associated with smoking, risk of depression, anxiety and stress, high risk of suicide and drug use.

**Conclusions:**

More than a third of students used social media excessively. This behavior was associated with negative health outcomes.

## Resumo

**Objetivo:**

Avaliar a prevalência do uso excessivo de redes sociais e identificar os seus fatores associados em estudantes do ensino médio do Sul do Brasil.

**Métodos:**

Trata-se de um estudo transversal de base populacional. Foram elegíveis para esta pesquisa todos os estudantes que estavam cursando o ensino médio no Instituto Federal do Rio Grande do Sul, campus Rio Grande, no 2º semestre de 2019. No total, 513 alunos participaram do estudo. A variável dependente deste estudo foi o uso excessivo de redes sociais, definido como mais de cinco horas por dia. Foram realizadas análises descritivas e bivariadas e, para verificar as associações, foi utilizada a regressão de Poisson, com ajuste robusto da variância.

**Resultados:**

A prevalência de estudantes que relataram uso excessivo de redes sociais foi de 35,9%. Os grupos mais suscetíveis a usar excessivamente as redes sociais foram: sexo feminino, cor de pele preta/parda e faixa etária entre 18 e 20 anos. Houve associação entre uso excessivo de redes sociais com tabagismo, risco de depressão, ansiedade e estresse, risco elevado de suicídio e uso de drogas.

**Conclusões:**

Mais de um terço dos estudantes usava em excesso as redes sociais. Esse comportamento esteve associado com desfechos negativos em saúde.

## INTRODUCTION

Social networks are a media option where users can create and consume content and share information in different formats, such as text, video, audio or photography.^1^ The use of this technology is a global phenomenon and more common among young people, a fact that raises concern among health professionals due to the negative influence on behavior, which can affect the biopsychosocial health of adolescents.^2,3^

The use of this type of media by teenagers is widespread. A survey conducted in 2018 in Brazil stated that about 82% of adolescents use and have a profile on social media, which corresponds to 22 million adolescent users.^4^

Excessive use of social media is defined in different ways in international studies, which use scales or time of use, and there is no cutoff point standardization in the literature. A survey conducted with adolescents in Singapore identified that longer exposure times per day on the internet—five hours or more—had greater associations with negative health outcomes in adolescents when compared to shorter periods.^5^

Positive implications, such as access to information, greater possibility of learning, establishing and maintaining relationships, ease of communicating feelings, identity formation and ease of receiving emotional support, can be linked to the use of social media as well.^1,6^ However, when use is excessive, it can cause direct damage to health.^7^ The main impacts of excessive use, more than two hours a day, include: changes in sleep pattern, attention and learning; higher incidence of obesity and depression; exposure to inappropriate, unsafe and inaccurate content.^1^ In addition, intemperate use can be harmful because it leads to content sharing and practices of bullying and cyberbullying, and dissemination of negative behaviors to others.^7^

The use of social media has intensified over the years, as a means to increase interactivity with daily life and culture. However, scientific evidence has not followed the pace of this change, and national and international studies on the subject are scarce. It should be noted that population surveys with adolescents carried out in Brazil have assessed screen time, but not specifically the use of social media, which reinforces the gap in literature.^8^ In this context, health professionals, managers, parents and teachers must understand the complexity of the topic, since the excessive use of social media can negatively affect the health and quality of life of adolescents. Therefore, this study aimed to assess the prevalence of excessive use of social media and identify any associated factors in high school students from southern Brazil.

## METHOD

The study was conducted based on a census of high school students at the Federal Institute of Rio Grande do Sul, campus Rio Grande (IFRS), Rio Grande do Sul (RS). IFRS is a federal educational institution that offers six technical courses integrated into secondary education, six subsequent high school courses and three higher education courses.

All students who were attending high school at IFRS, campus Rio Grande, in the second half of 2019, were eligible for this research, totaling 718 enrollments from the 1st to the 4th year. Individuals who were physically and/or cognitively unable to answer the questionnaire were excluded from the study. To collect data, we got in contact with the groups to present the research and hand the free and informed consent form to their guardians/parents (if under 18 years of age). Data was collected in September 2019, through self-administered questionnaires to students who agreed to participate. The questionnaire was applied by previously trained researchers, in a training course lasting 40 hours. They were responsible for identifying the students, distributing tablets, providing guidance and clarification on how to fill out the questionnaire. Students who chose not to participate in the research were treated as refusers and those who were not found in three visits were treated as losses.

The dependent variable was the overuse of social media, which was assessed by the question “how much time per day do you spend using social media (Facebook, Instagram, WhatsApp, Twitter, Snapchat or other)?” Those who reported five hours a day or more were considered to make excessive use of social media. The independent variables were: biological sex (female/male), age group in years (14–15, 16–17 and 18–20), skin color (white and black/brown), school year (1st, 2nd, 3rd and 4th), full-time study (no/yes), previous school failure (no/yes), maternal education (elementary, secondary and higher), economic level through principal component analysis, which encompassed nine variables related to household goods or household characteristics. The first component was extracted, which reached an eigenvalue of 3.00 and explained 33.4% of the variability of all components in tertiles (smallest, medium and largest). The following negative health conditions and behaviors were evaluated: unhealthy eating (through the dietary markers form of the Food and Nutritional Surveillance System - SISVAN),^9^ smoking (yes/no), alcohol use (yes/no), drug use (yes/no), high risk of suicide (measured using a self-administered version of the Mini-International Neuropsychiatric Interview),^10^ self-reported dissatisfaction with the body and risk of depression, anxiety and stress (measured by the Depression, Anxiety scale and Stress Scale).^11^

The data obtained were double-entered into the EpiData 3.1 software and later transferred to the Stata 15.1 statistical package, in which data analysis was performed. First, the sample was described according to independent variables, using absolute and relative frequency. Then, crude and adjusted analyses were performed to identify factors associated with excessive use of social media, using Poisson regression with robust adjustment of variance. The association between overuse of social media and risky behavior was made using the Fisher’s exact test. The significance level adopted was 5% for two-tailed tests.

This research project was approved by the Research Ethics Committee of Universidade Federal do Rio Grande, under opinion nº 128/2018, Certificate of Presentation for Ethical Appreciation (CAAE), 91281918.7.0000.5324. All individuals over 18 years of age or their guardians (if under 18) who agreed to participate in the research had to sign an informed consent form. Furthermore, before filling out the questionnaire, everyone should consent to participate in the survey.

## RESULTS

Among the 718 students enrolled, 84 had dropped out of the course at the time of data collection, totaling 634 students eligible to the sample. Of these, 25 refused to participate and 93 were not found at the time of data collection, which generated a response rate of 81.5%. Of 516 participants, three had no answers for the main variable of the study, resulting in a total of 513 individuals. Of these, 50.5% were males, 48.9% were between 16 and 17 years old, 77.1% were white and 37.2% were in the first year of school. Most of them studied full-time (60.9%) and 21.3% had previously failed school. Approximately half (49.4%) of the students reported a higher level of maternal education (Table 1).

**Table 1.** Description of high school students at the Federal Institute of Rio Grande do Sul, Rio Grande campus, 2019 (n=513).

	n	%
Biological sex ^b^		
Male	258	50.5
Female	253	49.5
Age group (years) ^d^		
14–15	97	19.1
16–17	249	48.9
18–20	163	32.0
Skin color ^c^		
White	393	77.1
Black/brown	117	22.9
School year		
1st	191	37.2
2nd	133	25.9
3rd	113	22.0
4th	76	14.8
Full-time study ^a^		
No	200	39.1
Yes	312	60.9
Previous school failure		
No	404	78.7
Yes	109	21.3
Maternal schooling ^f^		
Elementary school	66	13.6
High school	180	37.0
Higher education	241	49.4
Economic level (tertiles)^e^		
Low	169	33.3
Intermediate	168	33.2
High	170	33.5

^a^ *1 missing*; ^b^ *2 missing*; ^c^ *3 missing*; ^d^ *4 missing*; ^e^ *6 missing*; ^f^ *26 missing*.

The prevalence of students reporting excessive use of social media was 35.9% (confidence interval — 95%CI 31.7–40.0). Prevalence ranged from 28.3% for males and 48.7% for black people (Table 2). Practically all students (99.4%) used social media. Two thirds (66.0%) used them on cell phones and computers and 65.1% used them always or almost always before going to sleep.

**Table 2.** Crude and adjusted Poisson regression analyses and factors associated with excessive use of social media among high-school students at the Federal Institute of Rio Grande do Sul, Rio Grande campus, 2019 (n=513).

	Prevalence	Crude analysis	Adjusted analysis*
	%	PR (95%CI)	PR (95%CI)
Biological sex			
Male	28.3	1.00	1.00
Female	43.9	1.55 (1.22–1.97)	1.62 (1.27–2.06)
Age group (years)			
14–15	35.1	1.00	1.00
16–17	35.3	1.01 (0.73–1.39)	1.15 (0.82–1.63)
18–20	36.8	1.05 (0.75–1.47)	1.57 (1.00–2.46)
Skin color			
White	32.3	1.00	1.00
Black/brown	48.7	1.51 (1.19–1.91)	1.44 (1.13–1.83)
School year			
1st	40.3	1.00	1.00
2nd	32.3	0.80 (0.59–1.08)	0.68 (0.49–0.94)
3rd	36.3	0.90 (0.67–1.21)	0.81 (0.55–1.17)
4th	30.3	0.75 (0.51–1.10)	0.52 (0.32–0.86)
Full-time study			
No	37.0	1.00	1.00
Yes	35.3	0.95 (0.75–1.21)	0.88 (0.69–1.12)
Previous school failure			
No	35.2	1.00	1.00
Yes	38.5	1.10 (0.84–1.44)	1.01 (0.73–1.40)
Maternal schooling			
Elementary school	45.5	1.00	1.00
High school	37.8	0.83 (0.60–1.15)	0.89 (0.64–1.23)
Higher education	31.5	0.69 (0.50–0.96)	0.76 (0.54–1.07)
Economic level (tertiles)			
Low	37.3	1.00	1.00
Intermediate	39.3	1.05 (0.80–1.38)	1.19 (0.90–1.58)
High	31.8	0.85 (0.64–1.14)	1.04 (0.76–1.44)

*Poisson regression with robust adjustment for variance. Those variables with p-value less than 0.20 remained in the final model after adjustments for possible confounding factors. PR: prevalence ratio; 95%CI: 95% confidence interval.

In the crude analysis, the groups most likely to use social media excessively were: females (prevalence ratio — PR=1.55; 95%CI 1.22–1.97) and black/brown skin color people (PR=1.51; 95%CI 1.19–1.91). Children of mothers with higher education had a protection for the outcome (PR=0.69; 95%CI 0.50–0.96) (Table 2). After adjustment, females (PR=1.62 95%CI 1.27–2.06) and black/brown skin color people (PR=1.44; 95%CI 1.13–1.83) remained associated. In addition, students aged 18 to 20 years were more likely to overuse social media (PR=1.57 95%CI 1.00–2.46), while those who were attending the second (PR=0.68; 95%CI 0.49–0.94) and four years (PR=0.52 95%CI 0.32–0.86) were less likely. Maternal education lost the association (Table 2).

Those who reported using social media excessively were more likely to not follow a healthy diet (36.9%), to smoke (9.4%), to consume alcohol (56.8%), and to do drugs (14, 2%), having a high risk of suicide (23.9%), being dissatisfied with their body (51.1%) and presenting a higher risk of depression/anxiety/stress (27.9%) compared to who reported not using social media excessively (Table 3).

**Table 3.** Association of excessive use of social media with negative health conditions and behaviors among high-school students at the Federal Institute of Rio Grande do Sul, Rio Grande campus, 2019 (n=513).

Outcome	%	Excessive use of social media		
		No	Yes	p-value*
		% (95%CI)**	% (95%CI)**	
Unhealthy food	29.7	25.7 (20.9–30.5)	36.9 (29.7–44.0)	0.01
Tobacco use	6.3	4.6 (2.3–6.9)	9.4 (5.1–13.7)	0.04
Alcohol use	49.1	44.8 (39.4–50.2)	56.8 (49.6–64.1)	0.01
Drug use	10.4	8.2 (5.2–11.2)	14.2 (9.1–19.3)	0.05
High risk of suicide	17.2	13.4 (9.6–17.1)	23.9 (17.7–30.1)	<0.01
Body dissatisfaction	44.4	40.7 (35.4–46.1)	51.1 (43.8–58.4)	0.03
Risk of depression, anxiety and stress	19.8	15.2 (11.3–19.2)	27.9 (21.3–34.6)	<0.01

*Fisher’s exact test; **%: prevalence; 95%CI: 95% confidence interval.

**Figure 1.**
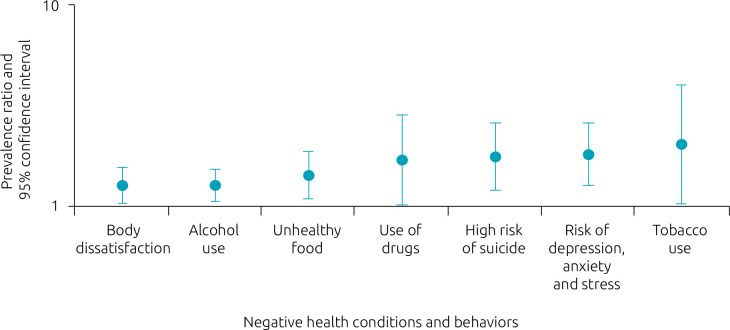
Association of excessive use of social media with negative health conditions and behaviors among high-school students at the Federal Institute of Rio Grande do Sul, Rio Grande campus, 2019 (n=513).

Figure 1 shows the association of excessive use of social media with negative health conditions and behaviors. The associations were significant for all outcomes, the strongest ones being with smoking (PR=2.05; 95%CI 1.05–4.01), risk of depression, anxiety and stress (PR=1.83; 95%CI 1 .29–2.60), high risk of suicide (PR=1.79; 95%CI 1.22–2.62) and drug use (PR=1.73; 95%CI 1.04–2.87).

## DISCUSSION

The present study evaluated the prevalence of excessive use of social media and identified associated factors among high school students in southern Brazil. We found that 35.9% of individuals used social networks excessively. Female, older individuals with black/brown skin color were more likely to overuse social media, and adolescents who were in the second and fourth years were less likely to presente this outcome. Smoking, risk of depression, anxiety and stress, increased risk of suicide and drug use were strongly associated with this behavior.

The estimated prevalence in this study was higher than what was found in other investigations on the subject. For example, the proportion of high school students who made excessive use of social media in a survey conducted in the Czech Republic in 2017 was 25.9%.^12^ However, the study considered it as “high use” of social media two hours or more per day. Using this same cutoff point for the outcome, a study in Canada from 2019 reported 27.6% of a sample of adolescents in elementary and high school with this behavior.^13^ However, in a survey conducted in six Europeans countries in 2014, the prevalence was slightly higher (38.8%).^14^ There are few studies in the literature that have estimated the prevalence of excessive use of social media in primary education students, and there are still differences in the cutoff point for the definition of the outcome, which makes comparability difficult.

Female adolescents were almost twice as likely to use social media excessively when compared to males, which is a consistent association in the literature on the subject.^9,15,16^ These differences can be explained by the different motivations for using social media between genders. Adolescents communicate more frequently with same-sex friends and family members and use social media to maintain friendships, because they like this form of social interaction. On the other hand, boys communicate more often with people they’ve never met and with same-sex friends about online games and sports, as well as use it to participate in groups.^15^

Black/brown students were also more likely to use social networks excessively, corroborating the study by Tynes and Mitchell conducted in the United States in 2014, with a representative sample of young people aged 10 to 17 years.^17^ Young black people were found to be more likely to use social networks, especially to talk to people they have met online and have not met in person, also being more likely to engage in sexual behavior online.^17^

Older individuals were more likely to overuse social media. However, first-year students had the highest prevalence for the outcome. In view of these contradictory findings, many of the students aged 18 to 20 years had previously failed school and were in their first year (data not shown), which could explain these results. Sampasa-Kanynga et al., investigating the associations between social media use, school connectivity, and academic performance in high school and elementary students from Ontario, Canada, found that heavy use of social media was negatively associated with school connectivity and academic performance.^13^ Such findings may help to better understand our findings, since the excessive use of social networking sites can affect school performance of adolescents.

The results also showed that participants who made excessive use of social networks were more likely to have an unhealthy diet and body dissatisfaction. This use is associated with greater chances of skipping breakfast and consuming energy drinks.^18^ It is possible that the increase in time on social media reduces the time to prepare or eat meals. Another explanation would be basically the food choices that young people are currently making.^18^ Body dissatisfaction, in turn, seems to be stimulated by increased exposure to—and comparison with—socially established ideal images.^19^ In a study with Brazilian adolescents, there was an almost five times greater risk of body dissatisfaction among those who reported using Instagram and Facebook 5 to 10 times a day.^20^ In addition, adolescents report feeling a pressure to post on social media in a way they seem perfect, selecting and editing details in their posts.^21^ This overexposure makes teenagers receive more feedback about their appearance, which can contribute to the increase in body dissatisfaction.^22^

Young people who used social media excessively were more likely to use alcohol, to smoke and to use illicit drugs. One mechanism that can explain this result is the influence of peers, as the probability of using certain substances is greater when the individual has a friend who also has this behavior.^23^ Through social media, social contacts with peers are no longer restricted to physical barriers. This can increase the exposure to content that encourages the use of alcohol, tobacco and illicit drugs. In addition, exposure in online environments can become more comfortable under alcohol use, which increases the perception of extroversion and popularity.^24^

In this study, young people who reported using social media excessively were more likely to be at risk of anxiety, depression and stress, as well as of suicide, associations that have already been identified in other research (in the United States in 2018 and in Canada in 2015) on the subject.^25,26^ Social networks are tools that allow great social interaction, but with superficial connections, not replacing face-to-face communication. This type of relationship can lead teenagers to feelings of loneliness and depression. The publication of messages with distorted content about success, material goods, good physical appearance, impressions of well-being and happiness on the part of virtual friends is recurrent. Continued exposure to this type of material can trigger feelings of incapacity or being out of bounds, which can trigger anxiety or depression in individuals.^27^ In addition, individuals who spend more time on social media are likely to be less involved in other activities of health promotion, which is negatively correlated with depressive symptoms.^25^ Finally, social networks are the main platform for cyberbullying,^1^ characterized as aggressive and intentional acts performed by an individual or group through electronic forms of contact.^28^ Victims of cyberbullying are more likely to attempt suicide than people who do not experience such situations.^29^

However, our results must be interpreted considering the study’s limitations and strengths. First, the cross-sectional design does not allow the establishment of temporality, which can lead to a reverse causality bias. Second, it is possible that the total time of use of social media was underestimated by the participants, as it is a measure of self-reported use. Third, the lack of standardization of a cut-off point for the excessive use of social networks made it difficult to compare studies, as each survey used a different one. One of the strengths to be highlighted in this study is the fact that it is a census in which everyone had the opportunity to participate, which reduces the interference of selection bias. In addition, an assessment of individual characteristics and possible health consequences of the excessive use of social media was carried out, with the identification of groups that are most vulnerable to it. This can contribute to the implementation of preventive measures such as educational interventions to raise awareness about the importance of reducing this behavior.

It is concluded that one third of high-school students in a city in southern Brazil used social media excessively. Older females, with black/brown skin color and who were at the beginning of high school were more likely to overuse social networks. The association of this use with behavioral characteristics of students was stronger for smoking, risk of depression, anxiety and stress, increased risk of suicide and drug use.
